# Determinants of felt demand for dengue vaccines in the North Caribbean region of Colombia

**DOI:** 10.1186/s12941-017-0213-1

**Published:** 2017-05-15

**Authors:** Yalil T. Bracho-Churio, Ruth A. Martínez-Vega, Alfonso J. Rodriguez-Morales, Ronald G. Díaz-Quijano, María L. Luna-González, Fredi A. Diaz-Quijano

**Affiliations:** 1 Organización Latinoamericana para el Fomento de la Investigación en Salud, Bucaramanga, Santander Colombia; 2 Public Health and Infection Research Group, Faculty of Health Sciences, Universidad Tecnológica de Pereira, Pereira, Risaralda Colombia; 3 Department of Epidemiology, Faculdade de Saúde Pública, Universidade de São Paulo, Av. Dr. Arnaldo, 715, São Paulo, SP CEP-01246-904 Brazil

## Abstract

**Background:**

The increasing burden associated with dengue in Latin America makes it essential to understand the community’s interest in acquiring vaccines, as an input to plan its introduction in endemic regions. The objective of this study is to learn the felt demand for dengue vaccines by estimating the willingness to pay and its associated factors in endemic communities of the North Caribbean region of Colombia.

**Methods:**

A population survey was administered from October to December 2015, including 1037 families in 11 municipalities in Colombia. One adult per family was interviewed on their perception and history of dengue. Participants received a description of four hypothetical scenarios of dengue vaccines, administered in a single dose or in 3 doses, with an effectiveness of 70% for 5 years or 95% for 30 years. The willingness to pay for each one of these vaccines was inquired vs. 5 hypothetical prices in Colombian pesos.

**Results:**

Most participants recognized dengue as a serious disease in children (99.3%) and adults (98.6%). 33 (3.2%) of the total respondents reported having suffered dengue and 19 (57.6%) of them required hospitalization. The price of the vaccine was inversely related to the willingness to pay. In addition, single dose vaccines (compared to 3 doses) and one with a protection of 95% for 30 years (compared to an effectiveness of 70% for 5 years), were associated with greater willingness to pay. Greater willingness to pay was observed among the respondents who considered it likely to get the disease, either themselves (OR 1.56; CI 95% 1.08–2.26) or their children (OR 1.89; CI 95% 1.28–2.81), in the next 5 years. The participants who have been diagnosed with dengue also showed greater willingness to pay (OR 1.89; CI 95% 1.01–3.54) compared to those who did not have this history.

**Conclusion:**

Factors such as price, number of doses and effectiveness can independently influence the decision to purchase a vaccine against an endemic disease, such as dengue. Additionally, this study reveals that background and perceptions of the disease can affect individuals’ interest in acquiring this type of preventive interventions.

## Background

Dengue continues to be the viral disease with the highest incidence transmitted by arthropods [1]. Recent estimates suggest that the annual incidence of dengue infections is 390 million, worldwide [1, 2]. In the Americas, dengue is a growing cause of morbidity and mortality, especially in the Caribbean region [3–5], representing a high burden in terms of disability-adjusted life years (DALYs) and associated costs [6]. In Colombia, dengue is a priority in public health, which in 2016 caused more than 100,000 reported cases with 195 associated deaths [7]. The Colombian Caribbean region has shown a tendency to rise and its departments, such as La Guajira, exhibit since 2011 incidence rates that exceed the country’s average [4].

Unfortunately, there is still no specific antiviral therapy and the current vector control seems to be insufficient to reduce the burden caused by dengue. Therefore, different vaccines are being developed with different levels of effectiveness and dosage schedules [8–11]. For example, CYD-TDC (Dengvaxia^®^) was the first vaccine to gain regulatory approval in some endemic countries. However, this vaccine requires a 3 dose regimen and showed only moderate efficacy (60%) in children and adolescents [12]. Other vaccines under evaluation would be potential alternatives with less dose schedules [13, 14]. Therefore, it is feasible that aspects such as the number of doses and the effectiveness of the vaccine are issues pertinent to the choice of a vaccine against dengue.

Given the increasing burden associated with dengue in the Americas [3], it is relevant to learn the felt demand of this intervention in the community, which serves as an input for the governments that must make decisions regarding prioritization of interventions for prevention and control. In addition, it is essential to understand the potential economic returns that the introduction of any vaccine would have [5]. Previous studies have been conducted in other populations evaluating the demand for vaccines through the estimation of willingness to pay [5, 15–19]. These studies have shown important differences among countries but few of them have evaluated the determinants of felt demand for dengue vaccines [17].

On the other hand, only one of the previous studies was developed in Colombia [5], specifically in Medellín, a city where only 0.1% of the population is self-recognized as indigenous and 6.2% as afro-descendant [20]. However, this demographic structure is very different from that observed in the Colombian Caribbean, where in addition to the ethnic differences there is a high socio-economic vulnerability.

Accordingly, the objective of this study is to learn the felt demand for dengue vaccines by estimating the willingness to pay and its associated factors in endemic communities of the North Caribbean region of Colombia.

## Methods

### Study setting

This research is part of a population-based cohort study (4). A population survey was administered from October to December 2015, including 1037 families selected through probabilistic sampling by conglomerates in eleven municipalities of three departments in Colombia. One adult from each family was interviewed on their perception and history of dengue. In addition, participants received a description of four scenarios of dengue vaccines, changing the effectiveness or the number of doses required in each scenario. The willingness to pay for each one of these vaccines was inquired vs. five hypothetical prices in Colombian pesos (COP).

### Study population and sampling technique

**Fig. 1:**
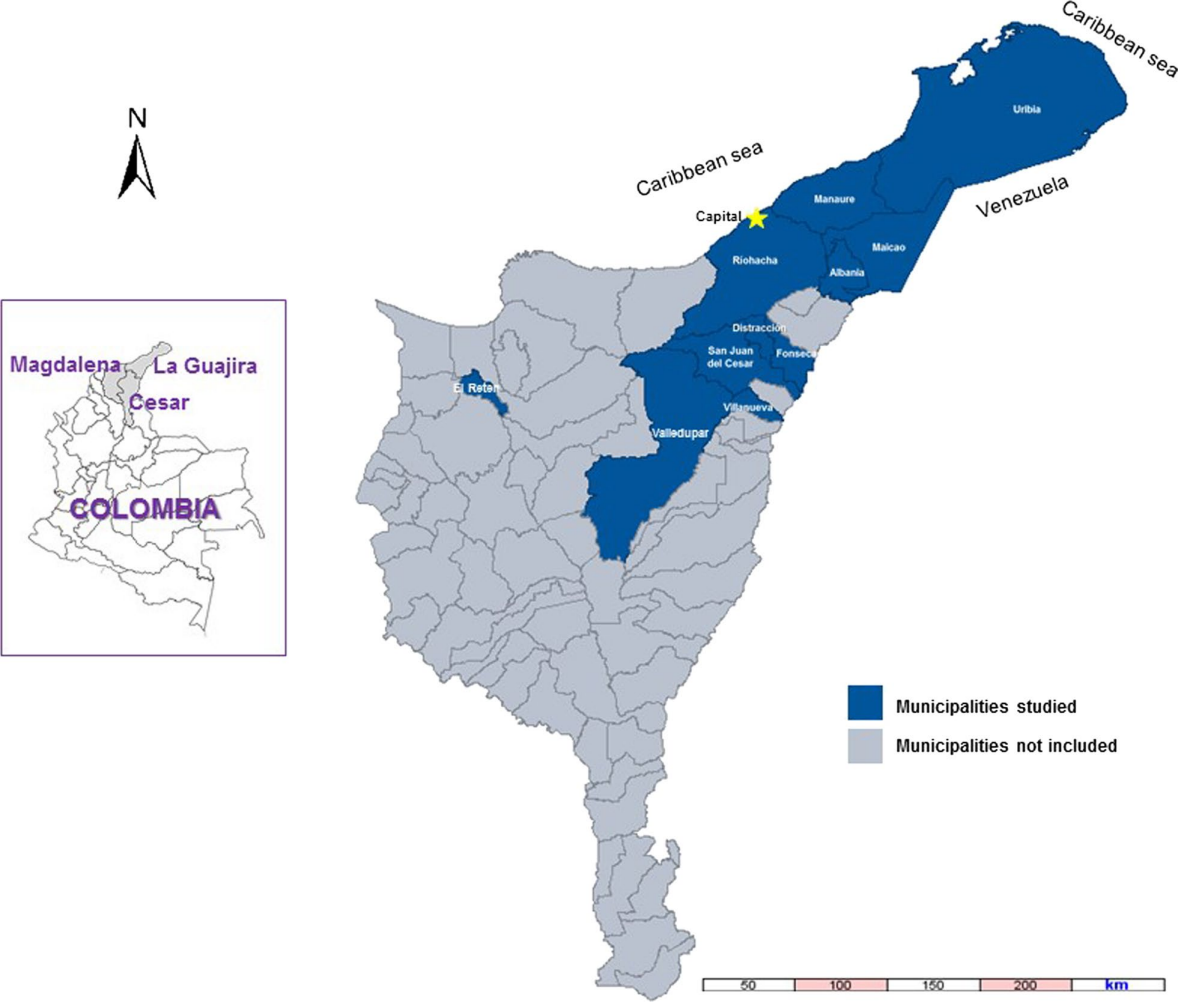
Relative location of the study sites in the North Caribbean region of Colombia

For this study, a sample was planned of at least 1000 families selected in 11 municipalities in the Colombian Caribbean. The municipalities included nine in the department of La Guajira (Riohacha, Albania, Fonseca, San Juan del Cesar, Distracción, Maicao, Villanueva, Uribia and Manaure) one in Cesar (Valledupar) and one in Magdalena (El Retén) (Fig. 1). Together these municipalities are estimated to have a total population of 1.3 million inhabitants [21]. Most of the surveys were administered in the Department of La Guajira (89.2%), in which approximately 42.4% of the population is self-recognized as indigenous and nearly 14% as afro-descendant [20].

The number of homes per municipality was assigned considering the size of the population, ranging from 20 families in El Retén (Magdalena) to 203 in Riohacha (La Guajira). Sampling by conglomerates was carried out for the selection of participants. Thus, blocks were selected in each municipality and on each block, following a mapping and population analysis, the number of homes was selected. Both selections were carried out at random. In each of the homes, residents were invited to participate and the questionnaire of this study was administered to the responsible adult who was at home at the time of the visit.

Only people in charge of the house at the time of the visit, who were at least 16 years old, were considered eligible to respond to the survey. People planning to change their place of residence in the next 6 months were excluded [4]. The respondents had not a dengue diagnosis at the time of the interview, but they may or may not have had the disease in the past [22]. In that sense, only 3.2% of the respondents reported a diagnosis of dengue in the past.

### Study procedure

Interviews were conducted by previously trained professional nurses, who carried out the informed consent process and the interview using a standardized questionnaire. This questionnaire was developed seeking to include the variables previously evaluated in a multi-country study for hypothetical vaccines with a 3 dose regimen [5]. In addition, we incorporated analogous scenarios for single-dose vaccines.

This questionnaire included questions on participants’ perception and experience regarding dengue. This was followed by a process of presentation of a scenario in which there is a vaccine with an effectiveness of 70% for 5 years, administered in 3 doses. This process included a graphical presentation of the concept of effectiveness as proposed by other authors for the respondent’s appropriate capacity to respond [5].

After that, participants were questioned on their willingness to pay for a vaccine for themselves with these characteristics, based on the following hypothetical prices: COP 3000, COP 60,000, COP 150,000, COP 300,000 and COP 900,000. These values are close to the following values in US dollars (USD) respectively (at the exchange rate of June 15, 2016): USD 1, USD 20, USD 50, USD 100 and USD 300. Subsequently, participants were asked if they would accept the same vaccine as provided freely. Then, the questionnaire of willingness to pay was repeated but presenting a scenario in which the vaccine would have an effectiveness of 95% for 30 years, administered in 3 doses. Then, these procedures were repeated for single-dose vaccines. Finally, the interviewers asked with a single question what is the average monthly income in the participant’s home. The value referred was recorded in COP.

### Statistical analysis

The analysis focused on modeling the willingness to pay as a binary variable. Number of doses, effectiveness and hypothetical price were considered as independent variables. Since the same person was providing information for a number of scenarios, logistic regression with the cluster option was used (applied to the participant) for the estimation of standard errors. The resulting model was evaluated in relation to goodness of fit using the Pearson and Hosmer–Lemeshow tests, with a confidence level of 95%.

This was followed by an exploratory analysis of the sociodemographic variables, perceptions and experiences of participants in relation to their intention to pay. The Odds Ratios (OR) were estimated for these analyses with 95% confidence intervals (CI 95%) for each of the responses, adjusting these estimates by the characteristics of the hypothetical vaccines. Variables with few data, for example those that were not applicable to the majority of the respondents or those categories with ten observations or less, were not considered for the multiple analysis. The Stata program (Version 11, 2009; College Station, TX) was used for the statistical analysis.

## Results

A total of 1037 individuals answered the survey and were included in the analysis, most of which were women (85%). Median age of participants was 42 years with an interquartile interval from 32 to 54. Regarding level of educational, 30.4% of participants had a basic primary level or lower, 43.9% had high school (either incomplete or graduated) and 25.7% had any superior education. On the other hand, referred income ranged between none and approximately COP 9 million (USD 3000) with a median of COP 600.000 (USD 200) and an interquartile interval from COP 300.000 to COP 800.000 (USD 100 to USD 267).

Almost all participants recognized dengue as a serious disease in children (99.3%), as well as in adults (98.6%). In addition, the majority of respondents considered it was likely they would get dengue in the next few years (Table 1). In turn, 33 respondents (3.2%) reported having previously had dengue (diagnosed by a physician) and 19 (1.84%) of them required hospitalization due to the virus. It is interesting that 15 respondents (1.45%) reported that a family member has died of dengue and 55 (5.33%) mentioned the death of a neighbor caused by dengue.

**Table 1:** Perceptions and experiences regarding dengue

Questions about dengue	(n)	Responses		
		Yes	No	Does not know/does not remember
Is dengue is a serious disease for children?	(1037)	1030 (99.3%)	4 (0.4%)	3 (0.3%)
Is dengue is a serious disease for adults?	(1037)	1022 (98.6%)	10 (1%)	5 (0.5%)
Are you likely to get dengue in the next 5 years?	(1034)	836 (80.9%)	88 (8.5%)	110 (10.6%)
Are your children likely to get dengue in the next 5 years?	(1032)	785 (76.1%)	78 (7.6%)	169 (16.4%)
Has a doctor ever diagnosed you with dengue?	(1037)	33 (3.2%)	998 (96.2%)	6 (0.6%)
Did you require hospitalization due to dengue?	(33)	19 (57.6%)	14 (42.4%)	
Has a doctor ever diagnosed anyone in your family with dengue?	(1035)	236 (22.8%)	798 (77.1%)	1 (0.1%)
Did the family member require hospitalization due to dengue?	(236)	206 (87.3%)	27 (11.4%)	3 (1.3%)
Has a family member ever died from dengue?	(1025)	15 (1.5%)	1003 (97.9%)	7 (0.7%)
Has a doctor ever diagnosed anyone in your neighborhood with dengue?	(1036)	238 (23%)	459 (44.3%)	339 (32.7%)
Has anyone in your neighborhood been hospitalized due to dengue?	(1037)	232 (22.4%)	437 (42.1%)	368 (35.5%)
Has anyone in your neighborhood died from dengue?	(1035)	55 (5.3%)	594 (57.4%)	386 (37.3%)
Have you ever been vaccinated against yellow fever?	(1037)	885 (85.3%)	81 (7.8%)	71 (6.8%)
Have you ever paid for a vaccine to prevent a disease?	(1033)	60 (5.8%)	960 (92.9%)	13 (1.3%)

In relation to vaccines, the majority of respondents (85.3%) claim to have received a dose of the yellow fever vaccine. Only 60 (5.81%) individuals claim to have paid for any vaccine. Other background information is presented in Table 1. According to the criteria of the interviewer, 92.2% (955/1036) of participants answered the questions related to effectiveness and duration of the effect of the vaccine adequately.

**Fig. 2:**
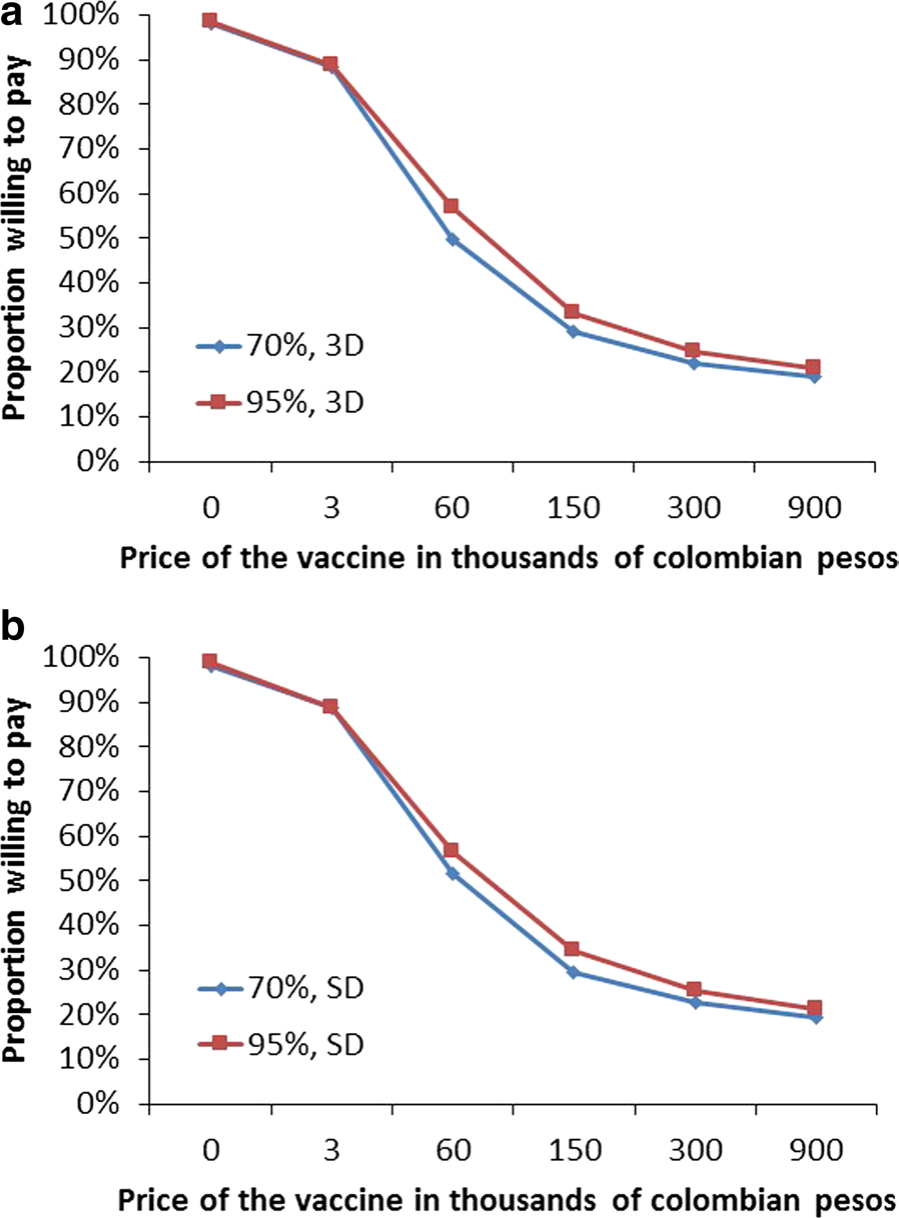
Willingness to pay for a vaccine against dengue, depending on the price of the vaccine and its effectiveness. **a** Vaccine administered in 3 doses (3D). **b** Vaccine administered in a single dose (SD). *Blue* shows the willingness to pay for a vaccine with an effectiveness of 70% and a duration of 5 years. *Red* shows the willingness to pay for a vaccine with an effectiveness of 95% and a duration of 30 years

The price of the vaccine was inversely related to the willingness to pay (Fig. 2). The vaccine with an effectiveness of 70% and a duration of 5 years, administered in 3 doses, had a willingness to pay of 88.6% when its value was COP 3000 (approximately USD 1); this percentage dropped to 51.8% when the value was COP 60,000 (≈USD 20); 29% with a value of COP 150,000 (≈USD 50); 22.1% when the value was COP 300,000 (≈USD 100); and, 18.9% when the value amounted to COP 900,000 (≈USD 300). However, when asked if they would accept the vaccine for free, the percentage of acceptance was 98.1%. This proportion of acceptance of the vaccine for free was similar for the other hypothetical vaccines (Fig. 2).

The willingness to pay for a vaccine with an effectiveness of 95% and a duration of 30 years, as well as a single-dose vaccine, showed a very similar tendency in relation to the price of the vaccine (Figs. 2a, b). However, the logistic regression model showed that, in addition to price, effectiveness and number of doses also had an impact on the willingness to pay (Table 2). Specifically, the vaccine with an effectiveness of 95% and a duration of 30 years had an odds 19% higher in the willingness to pay, compared with that of a 70% effectiveness and a duration of 5 years (OR 1.19; CI 95% 1.14–1.24).

**Table 2:** Association between the characteristics of the hypothetical dengue vaccine and the intention to pay for it

Characteristic	OR (CI 95%)	p value
Effectiveness of 95%^a^	1.19 (1.14–1.24)	<0.001
Value of the vaccine in COP^b^	1	
3000 (USD 1)	0.13 (0.09–0.2)	<0.001
60,000 (USD 20)	0.02 (0.013–0.03)	<0.001
150,000 (USD 50)	0.008 (0.005–0.012)	<0.001
300,000 (USD 100)	0.005 (0.003–0.008)	<0.001
900,000 (USD 300)	0.004 (0.003–0.007)	<0.001
3 dose (vs. single dose)	0.97 (0.95–0.99)	0.001

The values predicted by this model were similar to those observed according to the Pearson (p = 0.98) and Hosmer–Lemeshow (p = 0.99)

^a^In reference to an effectiveness of 95% for 30 years, compared to an effectiveness of 70% for 5 years

^b^The free category was used as the reference. Value in US dollars for 15 June 2016

In turn, the vaccine that requires 3 doses, would have an odds 3% lower of willingness to pay, compared to single-dose vaccines (OR 0.97; CI 95% 0.95–0.99). The model made up of the above variables presented a high goodness of fit, considering that the predicted values were similar to those observed according to the Pearson (p = 0.98) and Hosmer–Lemeshow (p = 0.99) tests. In addition, associations of this model were maintained when they were adjusted by municipality (data not shown).

Regarding sociodemographic variables, sex and age were not associated with the willingness to pay for a dengue vaccine. However, independently of the characteristics of vaccine (price, number of doses and effectiveness), willingness to pay was positively associated with level of education (Table 3). In relation to the monthly income mentioned by the respondent, we observed a positive association between this variable and the willingness to pay. Specifically, each increase of COP 300,000 (≈USD 100) in monthly income was associated with an 11% increase in the odds of paying for a dengue vaccine (Table 3).

**Table 3:** Sociodemographic factors, perceptions and experiences associated with the willingness to pay for a vaccine against dengue

Variable	OR^a^ (CI 95%)	p value
Sociodemographic		
Sex (female compared with men)	1.01 (0.76–1.34)	0.94
Age (years)	1 (0.99–1.01)	0.96
Level of education		
Basic primary or lower	1	
High school (graduated or incomplete)	1.56 (1.21–2.03)	0.001
Any superior education	2.05 (1.53–2.75)	<0.001
Income (every COP 300,000 ≈ USD 100)	1.11 (1.05–1.17)	<0.001
Perceptions		
Are you likely to get dengue in the next 5 years?		
No	1.0	
Yes	1.56 (1.08–2.26)	0.02
Does not know	1.27 (0.81–1.99)	0.30
Are your children likely to get dengue in the next 5 years?		
No	1.0	
Yes	1.89 (1.28–2.81)	0.001
Does not know	1.2 (0.78–1.86)	0.41
Experiences		
Has a doctor ever diagnosed you with dengue? (yes vs. no)	1.89 (1.01–3.54)	0.05
Has a doctor ever diagnosed anyone in your family with dengue? (yes vs. no)	1.16 (0.9–1.51)	0.25
Has a family member ever died from dengue? (yes vs. no)	1.26 (0.61–2.63)	0.53
Has a doctor ever diagnosed anyone in your neighborhood with dengue?		
No	1.0	
Yes	1.04 (0.79–1.37)	0.78
Does not know	0.64 (0.5–0.82)	<0.001
Has anyone in your neighborhood been hospitalized due to dengue?		
No	1.0	
Yes	0.96 (0.72–1.26)	0.75
Does not know	0.6 (0.47–0.77)	<0.001
Has anyone in your neighborhood died from dengue?		
No	1.0	
Yes	0.71 (0.44–1.15)	0.17
Does not know	0.54 (0.43–0.68)	<0.001
Have you ever been vaccinated against yellow fever?		
No	1.0	
Yes	1.82 (1.22–2.73)	0.003
Does not know	1.66 (0.95–2.89)	0.08
Have you ever paid for a vaccine to prevent a disease?		
No	1.0	
Yes	0.93 (0.6–1.45)	0.75
Does not know	0.18 (0.1–0.32)	<0.001

^a^Each OR is adjusted by the vaccine characteristics (price, number of doses and effectiveness)

On the other hand,, greater willingness to pay was observed among the respondents who considered it likely to get the disease, either themselves (OR 1.56; CI 95% 1.08–2.26) or their children (OR 1.89; CI 95% 1.28–2.81), in the next 5 years (Table 3). The participants who have been diagnosed with dengue also showed greater willingness to pay (OR 1.89; CI 95% 1.01–3.54) compared to those who did not have this history. In addition, participants who have been vaccinated against yellow fever also indicated greater willingness to pay for a dengue vaccine (OR 1.82; CI 95% 1.22–2.73).

Moreover, the responses indicated that the lack of knowledge of background information related to dengue were associated with lower odds to pay. Specifically, participants who mentioned they did not know whether they were diagnosed as having dengue (OR 0.17; CI 95% 0.05–0.54), or whether anyone in the neighborhood had been diagnosed (OR 0.64; CI 95% 0.5–0.82) or died from dengue (OR 0.54; CI 95% 0.43–0.68), showed a significantly lower odds of paying for the vaccine (Table 3). Finally, the individuals who said they did not know or could not remember having paid for any vaccines, also showed less willingness to pay, compared to those who responded negatively to the same question (OR 0.18; CI 95% 0.1–0.32).

## Discussion

Dengue continues to be a major global public health problem. In the study of the global burden of disease, an average of 9221 deaths from dengue per year was estimated between 1990 and 2013 [6]. The incidence increased significantly between 1990 and 2013, with a number of cases that multiplies each decade, from 8.3 million in 1990, to 58.4 million cases in 2013 [6]. Considering the fatal and non-fatal results as a whole, dengue was responsible for 1.14 million DALYs in 2013 [6]. In Colombia, it is a widely distributed disease that has caused epidemics over the past three decades (3), especially in the Colombian Caribbean, where the municipalities assessed in this paper are located [4].

For all the above reasons, there was therefore a marked interest in having dengue vaccines, both in endemic areas, as well as for travelers going to these regions, in order to reduce the impact of this burden. Decisions about introduction of a vaccine require careful assessment at the country level, including consideration of local priorities, dengue epidemiology, affordability and budget impact [23], as well as, the cost-effectiveness of dengue vaccination compared with other potential strategies [24].

In that sense, vaccine introduction must consider the felt demand in order to involve the community in the prioritization of specific interventions. In this context, economic evaluations, including the willingness to pay for vaccines, take on great importance and are related to the potential vaccination coverage, especially for those that will not be included in the national immunization programs but may be purchased by the population concerned [11].

The results of this study reveal aspects, such as the fact that the number of doses and the effectiveness of the vaccine are associated with the community’s willingness to pay. These associations suggest that the population studied could be less interested in the CYD-TDC (Dengvaxia^®^) because this vaccine, besides requiring 3 doses, have exhibited only a 60% of effectiveness [12], which is lower than those hypothetical scenarios evaluated in our study.

Regarding the price, this study shows that the median willingness to pay is about COP 60,000 (approximately USD 20). Higher values would make more than half of the population decline the immunization (Fig. 2). In similar studies, the median willingness to pay in Vietnam was USD 26, USD 70 in Thailand and USD 23 in a district of Medellin, Colombia, where 400 families were evaluated [5], which is very similar to our results and consistent for the country.

On the other hand, the median willingness to pay in our study was higher than that observed in Indonesia for an hypothetically safe and fully protective dengue vaccine, which was about USD 4 [17]; but it was lower than that reported in Brazil for the CYD-TDC (Dengvaxia^®^), which was about USD 33 [15]. This indicates that the estimates of this study are within the range observed in the literature.

These results, as in previous studies [5], suggest that respondents took the hypothetical scenario of buying a dengue vaccine seriously. They also suggest the possibility that there is a market for dengue vaccines and that sales can be robust if prices are lower than the estimated median of our study, which in the case of Colombia included a greater number of households assessed than the previous study [5]. However, it is important to mention that despite the willingness to pay, similar to that reported in other dengue studies, less than 6% of subjects evaluated claimed to have paid for some vaccine in their life.

Our study identified several associations between willingness to pay and factors such as the educational level, income, perceptions and previous experiences of the interviewee. Although evaluated, these associations were not statistically significant in a recent study in Indonesia [17]. This was probably due to that study having a smaller sample size. However, the aforementioned study found that the willingness to pay is associated with good attitudes and preventive practices of the population. The joint evidence from these studies indicates that the felt demand for a dengue vaccine will be conditioned by multiple factors of the population, including the educational level, the economic situation, the experience with the disease, and the way in which people are currently facing this arbovirosis.

This type of willingness-to-pay studies may have significant limitations. For example, to identify the maximum amount of money that a respondent would be willing to pay, the interviewers went through a list of vaccine prices in an ascending manner starting from a cheapest vaccine bid. This might have caused an anchoring effect bias and therefore a risk of underestimating the price which participants would pay [17]. In the present study, the risk of this bias could be reduced because the free vaccine scenario was offered at the end for each type of vaccine.

On the other hand, it is possible that other types of bias affect respondents’ responses. For example, desirability bias in which participants might tend to give favorable answers; and/or, the “hypothetical bias”, in which participants misstate their actual preferences in a hypothetical survey compared to a real-life situation [17]. Indeed, it is difficult to know the direction of the joint effect of all potential biases on estimation. However, we consider that possible biases would not invalidate the associations found between the willingness to pay and the determinants identified.

As another limitation, this study does not refer to any particular available vaccine. In fact, it could be considered that the scenarios raised exclude vaccines with effectiveness lower than 70% or those designed to apply in two doses [8, 11]. However, the purpose of this study was to understand vaccine demand, which was performed in an objective way in a country where there is no supply of any dengue vaccine. Thus, this study allowed us to identify determinants of the demand for vaccines that could even be extrapolated by analogy to other diseases. These determinants included characteristics of vaccines (dose, effectiveness and price), as well as, epidemiological factors such as previous exposure to the disease and the perception of risk.

For example, participants who believe that they are likely to get the disease and those who have been diagnosed as having it, showed greater willingness to pay. In another sense, patients who are unaware that there has been morbidity or mortality from dengue in their environment, showed less interest in buying a vaccine. Therefore, it is feasible that education in dengue and its epidemiology can raise community awareness and increase the demand for preventive interventions.

All this suggests that the intention to pay can be considered a good indicator of the felt demand for vaccines, since it is closely related to the perception of risk and personal experiences. So it is expected that the felt demand for vaccines is greater in areas with higher morbidity and mortality rates. Therefore, it could be expected that other endemic municipalities, not specifically included in this evaluation, have similar values based on the epidemiological importance of dengue therein.

In addition, aspects such as having been vaccinated for yellow fever was associated with a greater willingness to pay, suggesting greater awareness of the benefit of immunization, which may be associated with positive reinforcement, low frequency of adverse effects and access to health services, among other factors. In addition, the relationship between monthly income and the dependent variable is consistent and validates the consistency of the questionnaire, as was to be expected because individuals with better incomes tend to be more willing to pay for a vaccine.

Another relevant aspect in the region is the co-circulation of other viruses transmitted by the same vector, such as Chikungunya and Zika, which have taken on great importance over the last year [25, 26]. This epidemiological phenomenon can reinforce community awareness of the arboviruses [27, 28] in general and, thus, positively reinforce the willingness to pay for dengue vaccines [16]. Secondly, this study also showed an apparent coverage of self-referenced vaccination for yellow fever in the region of study of more than 85%. This is particularly important at a time when yellow fever is the subject of global concern due to epidemics in Africa and other regions of the world [29, 30].

The increase in the number of dengue cases and the lack of vaccines against arboviruses leads governments to take into account several types of effective means to control the disease [31]. Similar to previous studies [5, 15, 17], this paper provides important information on the number of individuals in endemic areas who are willing to pay for a dengue vaccine to avoid the risk of getting the infection, perhaps especially those who have only had a previous infection by one serotype and want to avoid further infection by other serotypes.

The results of this study can be used in a cost-benefit analysis to plan an appropriate introduction of dengue vaccines. Some studies have already used information generated in this regard [32], where with a theoretical efficiency of 70%, a potential effect of herd immunity can even be reached with a vaccination coverage of 82%. At these values, the dengue vaccine could be cost-effective for costs of vaccination of up to USD 534 per individual vaccinated, saving up to USD 204. It is suggested that even at lower effectiveness rates, they are still cost-effective [32].

These estimates, which are consistent with those of previous studies in Colombia and in Asia [5, 16, 18], present an approach that can be associated with the impact that they can also have on the reduction of the disease. The results can be incorporated in the analysis of prioritization of different health interventions on the national level [5]. Moreover, studies such as the one carried out could be suggested to health authorities to be conducted at the national level in municipalities prioritized by their epidemiological characteristics in the country.

The study can also help decision makers understand how a large part of the population in endemic areas, can be covered by the subsidy of dengue vaccines in the implementation of campaigns on the national level and contribute to the design of public vaccination policies, which should definitely be integrated with other preventive strategies to control dengue in the country [10], and even more so since new vaccines show a better effectiveness and safety profile [33]. On the other hand, knowledge about contextual factors determining willingness to pay are essential to design vaccine introduction programs [34].

In conclusion, this study shows evidence, for the first time for the Colombian Caribbean region, of how factors such as price, number of doses and effectiveness can independently influence the decision to purchase a vaccine against an endemic disease, such as dengue. Additionally, this study reveals how community background and perceptions of the disease can affect individuals’ interest in acquiring this type of preventive interventions.

## Abbreviations

COP: Colombian pesos
USD: US dollars
OR: odds ratio
CI 95%: confidence intervals of 95%
3D: 3 doses
SD: single-dose
